# Whole-Exome Sequencing Identified a Novel Compound Heterozygous Mutation of* LRRC6* in a Chinese Primary Ciliary Dyskinesia Patient

**DOI:** 10.1155/2018/1854269

**Published:** 2018-01-08

**Authors:** Lv Liu, Hong Luo

**Affiliations:** Department of Respiratory Medicine, Diagnosis and Treatment Center of Respiratory Disease, The Second Xiangya Hospital, Central South University, Changsha, Hunan 410011, China

## Abstract

Primary ciliary dyskinesia (PCD) is a clinical rare peculiar disorder, mainly featured by respiratory infection, tympanitis, nasosinusitis, and male infertility. Previous study demonstrated it is an autosomal recessive disease and by 2017 almost 40 pathologic genes have been identified. Among them are the leucine-rich repeat- (LRR-) containing 6 (LRRC6) codes for a 463-amino-acid cytoplasmic protein, expressed distinctively in motile cilia cells, including the testis cells and the respiratory epithelial cells. In this study, we applied whole-exome sequencing combined with PCD-known genes filtering to explore the genetic lesion of a PCD patient. A novel compound heterozygous mutation in* LRRC6* (c.183T>G/p.N61K; c.179-1G>A) was identified and coseparated in this family. The missense mutation (c.183T>G/p.N61K) may lead to a substitution of asparagine by lysine at position 61 in exon 3 of* LRRC6*. The splice site mutation (c.179-1G>A) may cause a premature stop codon in exon 4 and decrease the mRNA levels of* LRRC6*. Both mutations were not present in our 200 local controls, dbSNP, and 1000 genomes. Three bioinformatics programs also predicted that both mutations are deleterious. Our study not only further supported the importance of LRRC6 in PCD, but also expanded the spectrum of* LRRC6* mutations and will contribute to the genetic diagnosis and counseling of PCD patients.

## 1. Introduction

Primary ciliary dyskinesia (PCD; MIM: 244400) is an infrequent autosomal recessive disorder caused by disabled cilia structure or function and has a prevalence of 1 in 15,000–20,000 individuals [1, 2]. In clinic, patients who suffer from PCD is characterized by acute or chronic respiratory infection, tympanitis, nasosinusitis, and male infertility [3, 4]. Approximately 50% of the PCD patients may present laterality defects, such as dextrocardia or situs inversus totalis, called Kartagener's syndrome [5]. At present, nearly 40 PCD-etiological genes which encode the ciliary dynein or the regulatory factors have been reported [6]. However, due to crucial genetic heterogeneity and complexity of a diverse genetic architecture, the numbers of PCD-causing genes are much less than candidate genes [2, 4]. In addition, there are several new pathogenic genes such as* PIH1D3*,* DNAJB13*, and* TTC25 *having been gradually reported in PCD patients in recent years [7–10].

Leucine-rich repeat- (LRR-) containing 6 (LRRC6) is a cilia-related gene codes for a 463-amino acid cytoplasmic protein, which is distinctively expressed in motile cilia cells, including the testis cells and the respiratory epithelial cells [11]. As a PCD-pathologic gene,* LRRC6* was first identified in 2012 and so far 15 mutations have already been found [11, 12]. Animal experiment also indicates that the absence of LRRC6 can affect the motility of motile cilia, while morbigenous mutations in the* LRRC6* gene may affect motion of sperm flagella and motile cilia on respiratory tract and ultimately result in sperm movement obstacle, airway secretion removal disorders, and so forth [13, 14].

In this study, we employed whole-exome sequencing in combination with PCD-related genes list filtering to explore the genetic lesion of a patient with obvious PCD phenotypes including recurrent sinusitis, chronic bronchiectasis, and male infertility. A compound heterozygous mutation (c.183T>G/p.N61K; c.179-1G>A) of* LRRC6* was identified and cosegregated with the affected members in his family. Both mutations (c.183T>G/p.N61K; c.179-1G>A) of* LRRC6* were first reported worldwide and absent from the 1000 genomes, dbSNP144, and 200 local normal controls. Functional analysis revealed that both variants were deleterious. The Real-Time qPCR analysis showed that the mRNA expression levels of* LRRC6* in the proband were decreased significantly compared with other controls, indicating the consequence of the compound heterozygous mutation.

## 2. Methods

### 2.1. Patients and Subjects

The Review Board of The Second Xiangya Hospital of Central South University in China approved this research protocol, and informed consent was provided by each participant. All members in the family were enrolled and blood samples were obtained from the affected proband and the other family members. Subjects were reviewed based on their medical records and via CT scans of the lung and sinus.

### 2.2. Whole-Exome Sequencing

The genomic DNA was extracted by DNeasy blood and tissue kit (QIAGEN # 69506). And the exome capture, high throughput sequencing, and common filtering were performed in the Novogene Bioinformatics Institute (Beijing, China). All the exomes were captured by Agilent SureSelect Human All Exon V6 kits and sequenced by Illumina HiSeq X-10 platform. The strategies of data filtering are as follows as we have described [15, 16]: (1) variants in the 1000 Genomes Project (1000G, http://www.1000genomes.org) with MAF > 0.01 were excluded. (2) Variants in the dbSNP144 (https://www.ncbi.nlm.nih.gov/projects/SNP/) with MAF > 0.01 were also excluded. (3) The remaining data were filtered by PCD-related genes (Table S1). (4) Bioinformatics analysis was used for the remaining variants.

### 2.3. Mutation Validation and Cosegregation Analysis

Sanger sequencing by ABI 3130 DNA analyser was used to validate the candidate variants found in the whole-exome sequencing. Segregation analysis was performed in all family members [17, 18]. Primer pairs used to amplify fragments encompassing individual variants were designed by Primer 5 and the sequences of PCR primers will be provided upon request.

### 2.4. RNA Extraction and Real-Time qPCR

Total RNA was extracted by the PureLink® RNA Mini (Thermo Fisher Scientific, #12183025) from the peripheral blood in the proband and other controls. cDNA was synthesized from a total of 1 *μ*g of RNA using the RevertAid First Strand cDNA Synthesis Kit (Thermo Fisher Scientific, #K1621) with oligo (dT) primers. Real-Time qPCR reactions were carried out in Fast 7500 Real-Time PCR Systems (Applied Biosystems) using Maxima SYBR Green/ROX qPCR Master Mix (2x) (Thermo Fisher Scientific, #K0221). And 2^(−ΔΔCt)^ was used to analyze the comparative* LRRC6* mRNA expression levels between mutation group and healthy group. Each assay was performed in five independent tests. The data were analysed by unpaired two-tailed *t*-tests using Graph Pad Prism V.5 software (V.5.0). And the sequences of PCR primers will be provided upon request.

## 3. Results

### 3.1. Clinic Data

**Figure 1:**
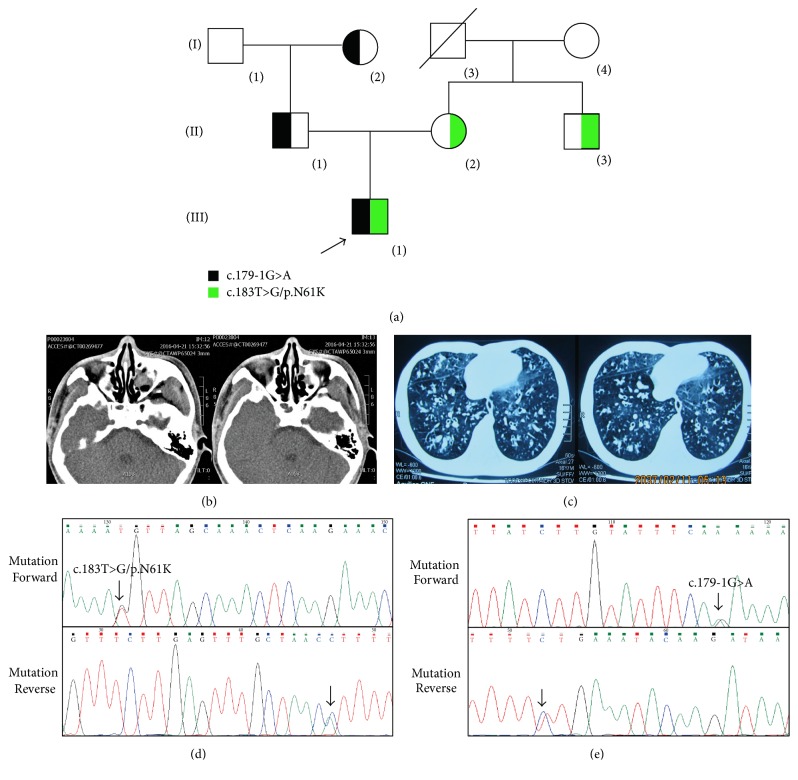
The clinic and genetic data of the proband. (a) Pedigree of the family affected with PCD. Squares indicate male family members; circles, female members; arrow, proband. (b) Sinus Computed Tomography (CT) revealed chronic sinusitis in the proband. (c) CT scans of the lung presented diffuse bronchiectasis of the proband. (d) Sequencing results of c.183T>G/p.N61K mutation in* LRRC6*. (e) Sequencing results of c.179-1G>A mutation in* LRRC6*.

A Chinese patient expressing typical symptoms and signs of PCD including repeatedly sinusitis, chronic bronchiectasis, and male infertility participated in this research (Figures 1(a), 1(b), and 1(c)). The patient (III-1), a 33-year-old male from Hunan Province in Central-South China, had a history of chronic cough, expectoration, recurrent nasal obstruction, and rhinorrhea from his childhood. He was also diagnosed with male infertility but lacked the manifestation of situs inversus totalis or* Heterotaxis*. Sinus Computed Tomography (CT) revealed chronic sinusitis (Figure 1(b)). The test of nasal nitric oxide concentrations was 29 ppb, which is lower than the PCD-specific nNO cutoff value (287 ppb). CT scans of the lung presented diffuse bronchiectasis (Figure 1(c)). As the other family members have none clinical features, the patient is considered as a sporadic PCD patient.

### 3.2. Genetic Analysis Identified a Novel Compound Heterozygous Mutation in LRRC6

Whole-exome sequencing yielded 10.13 Gb data with 99.82% coverage of target region and 99.1% of target covered over 10x. After alignment and single nucleotide variant calling, 54,282 variants were identified in the proband. We then performed the data filtering as we formerly described in Methods. Approximately 524 single nucleotide variants and indels were picked out. Next, we used the PCD-related genes list to filter the remaining variants, and a set of 5 variants in 4 genes were identified (Table 1). Bioinformatics analysis by MutationTaster, Polyphen-2, and SIFT was also carried out [19].

**Figure 2:**
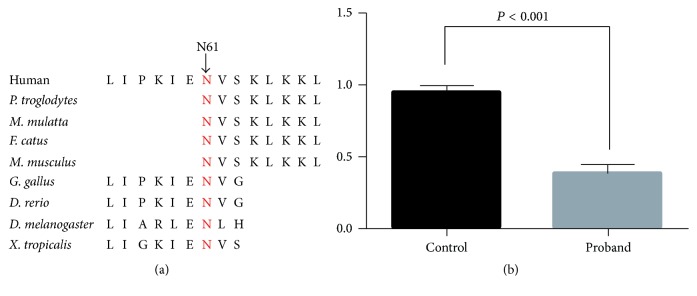
The analysis of two mutations in* LRRC6*. (a) Alignment of multiple LRRC6 protein sequences across species. The N61 affected amino acid locates in the highly conserved amino acid region in different mammals (from Ensembl). Red column shows the N61 site. (b) RNA expression of* LRRC6* affected the proband and controls. Mean expression (±SEM) of LRRC6 in the proband and control measured by Real-Time qPCR.

Sanger sequencing and bioinformatics analysis indicated that a novel compound heterozygous mutation (c.183T>G/p.N61K; c.179-1G>A) of* LRRC6* cosegregated with the affected family members (Figures 1(d) and 1(e)). The novel missense mutation (c.183T>G/p.N61K) was located in the highly conserved site in exon 3 of* LRRC6*, resulting in a substitution of asparagine by lysine (Figure 2(a)). The novel splice site mutation (c.179-1G>A) may induce the nonsense-mediated mRNA decay of* LRRC6*. Both novel mutations were also not found in our 200 local control cohorts [20].

### 3.3. The Novel Splice Site Mutation Decreases the mRNA Expression Level of LRRC6

The bioinformatics program MutationTaster predicated that both mutations were disease causing, because the splice site mutation (c.179-1G>A) of* LRRC6* may lead to a premature stop codon in exon 4. According to nonsense-mediated mRNA decay theory, the levels of* LRRC6* mRNA expression in affected patients may decrease. We then isolated the mRNA from peripheral blood in the proband and other controls. Real-time qPCR regarded the other controls levels of mRNA in* LRRC6* as “1.” The results revealed that the level of* LRRC6* mRNA expression was decreased significantly in the proband compared with the controls (Figure 2(b)) (*P* < 0.001).

## 4. Discussion

In this study, we combined whole-exome sequencing with PCD-related gene filtering to detect the potential genetic etiological factor in a typical Chinese PCD patient who was diagnosed as recurrent sinusitis, chronic bronchiectasis, and male infertility. A novel compound heterozygous mutation of* LRRC6 *(c.183T>G/p.N61K; c.179-1G>A) which was determined may underlie this patient. The novel missense mutation (c.183T>G/p.N61K) was located in the highly conserved domain of LRRC6 and resulted in a substitution of asparagine by lysine which may disturb the structure of LRRC6. The novel splice site mutation (c.179-1G>A) may decrease the mRNA levels of* LRRC6*, which have been further confirmed by Real-Time qPCR. Our study is consistent with previous reports that compound heterozygous mutation or homozygous mutations in LRRC6 may lead to PCD [11, 12, 14].

LRRC6 assembles Dynein Axonemal Assembly Factor 1 (DNAAF1) in structure, location, and function; both contain an LRR solenoid that consists of 6 leucine-rich repeats (LRRs) and an LRR cap occurring at the C-terminal to leucine-rich repeats [11, 21]. The LRR region in LRRC6 most closely resembles that of the SDS22-like subfamily of LRR proteins, a set of proteins with diverse functions, including splicing factors and nuclear export proteins [11]. Mutation in this domain may affect the beta-alpha unit existing in each leucine-rich repeat and have an impact on protein function by conformational change [14, 22]. The novel missense mutation (c.183T>G/p.N61K) may disturb the organization of LRRs motif and affect the movement of cilia [23]. The novel splice site mutation (c.179-1G>A) may lead to a premature stop codon in exon 4 and induce the nonsense-mediated mRNA decay of* LRRC6*. The decreased mRNA levels of* LRRC6* may affect its function in dynein arm assembly and building for cilia motility [11]. These two mutations inherited in a person at the same time may lead to PCD in the end.

*LRRC6* is a cilia-related gene distinctively expressed in motile cilia cells, including the testis cells, the respiratory epithelial cells, and the embryonic nodal cells [13]. It may play a role in dynein arm assembly and is hence essential for proper axoneme building for cilia motility [24]. The LRRC6 can transport the outer dynein arms protein from the cytoplasm to the cilia, and the loss-of-function and decrease of LRRC6 may affect the function of cilia. In addition, some research also revealed that LRRC6 can interact with ZMYND10, another PCD-causing gene, regulating the expression of the axonemal protein components DNAH5 and DNALI1 from respiratory cilia [25, 26]. So, the identification of mutations in* LRRC6* can provide us with more important information in researching the function role of LRRC6 in cilia. But now, there are only fifteen mutations having been detected in PCD-related patients. Our study further confirmed another two novel mutations (c.183T>G/p.N61K; c.179-1G>A) of* LRRC6 *in a Chinese PCD patient by whole-exome sequencing. The splice site mutation which was confirmed may decrease the mRNA levels of* LRRC6*, and the missense mutation may disturb the structure and function of LRRs domain in LRRC6. In this domain, a similar mutation (p.A74P) has been reported but indicated causing a milder PCD phenotype [11].

In conclusion, using whole-exome sequencing in combination with PCD-related genes list filtering strategy, we identified a newly compound heterozygous mutation in* LRRC6 *(c.183T>G/p.N61K; c.179-1G>A) in a Chinese PCD patient. Real-Time qPCR also revealed that the levels of* LRRC6* mRNA expression were decreased obviously. Our study not only further supported the important role of LRRC6 in PCD, but also expanded the spectrum of* LRRC6* mutations and will contribute to the genetic diagnosis and counseling of patients with PCD.

## Figures and Tables

**Table 1:** Variants identified by whole-exome sequencing in combination with PCD-related gene-filtering of the family.

Chr	POS	RB	AB	Gene name	AA change	MutationTaster	Polyphen-2	SIFT
8	133669149	A	C	LRRC6	NM_012472:exon3:c.T183G:p.N61K	Disease causing (0.99)	Damaging (0.999)	Damaging (0.00)
8	133669154	C	T	LRRC6	. NM_012472:exon4:c.179-1G>A		-	-
10	28229527	G	T	ARMC4	NM_001290021:exon6:c.C526A:p.P176T,ARMC4:	Polymorphism (0.99)	Damaging (0.99)	Tolerated (0.60)
16	70942236	A	G	HYDIN	NM_001270974:exon49:c.T8315C:p.F2772S	Disease causing (0.99)	Damaging (1)	Damaging (0.001)
17	78064076	GAAC	G	CCDC40	NM_001243342:exon18:c.2972_2974del:p.991_992del	Polymorphism (0.99)	-	-

CHR = chromosome; POS = position; RB = reference sequence base; AB = alternative base identified.

## Supplementary Materials

Supplementary Materials Table S1: the gene list of PCD-related gene.
