# Supplementary material for: Whole-Exome Sequencing Identified a Novel Compound Heterozygous Mutation of LRRC6 in a Chinese Primary Ciliary Dyskinesia Patient
Source: Biomed Res Int. 2018 Jan 8;2018:1854269. doi: 10.1155/2018/1854269 (PMC5817365; doi:10.1155/2018/1854269)
Supplement: Supplementary Materials — Table S1: the gene list of PCD-related gene. [file 1854269.f1.pdf]

Table S1. The gene list of PCD-related gene.

Gene Name							
DNAH5	CCDC114	DNAAF2	DYX1C1	RSPH3	CCDC164	MCIDAS	DNAH6
DNAI1	ARMC4	DNAAF3	SPAG1	RSPH4A	CCDC65	OFD1	PIH1D3
DNAI2	CCDC151	HEATR2	CCDC103	RSPH9	DNAH11	RPGR	DNAJB13
DNAL1	TTC25	LRRC6	C21orf59	CCDC39	HYDIN	DNAH8	SKT36
NME8	DNAAF1	ZMYND10	RSPH1	CCDC40	CCNO	GAS8	PRH1D3
